# Partial duplication of the anterior communicating artery: A case report

**DOI:** 10.1016/j.radcr.2025.04.061

**Published:** 2025-05-12

**Authors:** Hideki Endo, Hidetoshi Ono, Toshiichi Watanabe, Hirohiko Nakamura

**Affiliations:** a Department of Neurosurgery, Nakamura Memorial Hospital, South 1, West 14, Chuo-ku, Sapporo, Hokkaido 060-8570, Japan; b Department of Radiology, Nakamura Memorial Hospital, South 1, West 14, Chuo-ku, Sapporo, Hokkaido 060-8570, Japan

**Keywords:** Anterior cerebral artery, Anterior communicating artery, Digital subtraction angiography, Duplication, Magnetic resonance angiography, Partial duplication

## Abstract

Anatomical variations in the anterior cerebral artery and/or anterior communicating artery complex are common. However, partial duplication is a rare variant of the anterior communicating artery. To the best of our knowledge, there are only a few reports describing a partially duplicated anterior communicating artery. We report a case of a partially duplicated anterior communicating artery associated with an intracranial aneurysm, which was diagnosed by 3-Tesla magnetic resonance angiography and digital subtraction angiography. A 43-year-old woman was admitted to our hospital for postoperative examinations following coil embolization of an unruptured right internal carotid artery aneurysm performed 1 year earlier. Magnetic resonance angiography and digital subtraction angiography revealed no recurrence of the treated aneurysm, and a partially duplicated anterior communicating artery—there were 2 separate anterior communicating arteries on the right side and one on the left side. Digital subtraction angiography under contralateral carotid artery compression showed no aneurysm in the anterior communicating artery complex. This report provides additional evidence of partially duplicated anterior communicating arteries associated with an intracranial aneurysm. Careful imaging assessment is important to identify this rare anatomical variant, as well as other concomitant variations and associated aneurysms.

## Introduction

Anatomic variations in the anterior cerebral artery (ACA) and/or anterior communicating artery (ACoA) complex are common [[1], [2], [3]]. ACoA duplications can be mistaken for fenestrations and require careful observation [1,4]. Among these, the ACoA partial duplication is rare, with only a limited number of reports [2,4,5]. Herein, we report a case of a partially duplicated ACoA diagnosed by magnetic resonance angiography (MRA) and digital subtraction angiography (DSA).

## Case report

**Fig. 1:**
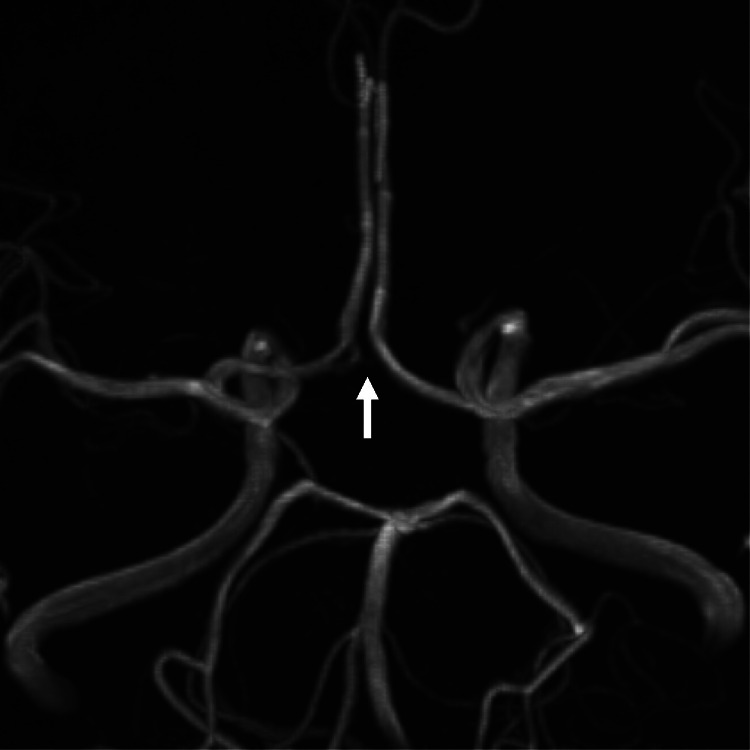
Magnetic resonance angiography showing partial duplication of the anterior communicating artery (arrow).

**Fig. 2:**
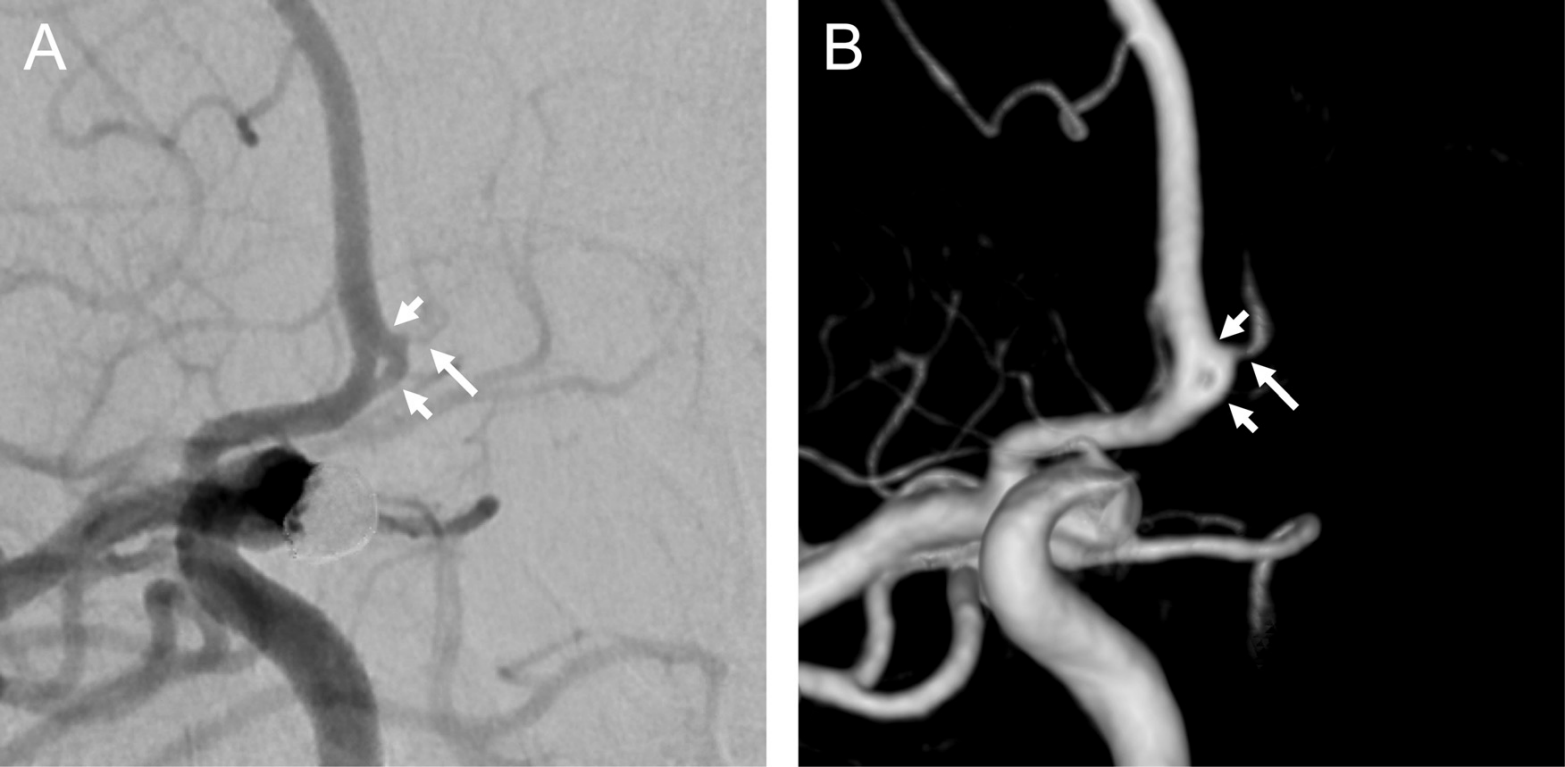
Right internal carotid angiography showing partial duplication of the anterior communicating artery (A, 2-dimensional image; B, 3-dimensional image). There were 2 separate anterior communicating arteries on the right side (short arrows) and one on the left side (long arrow).

**Fig. 3:**
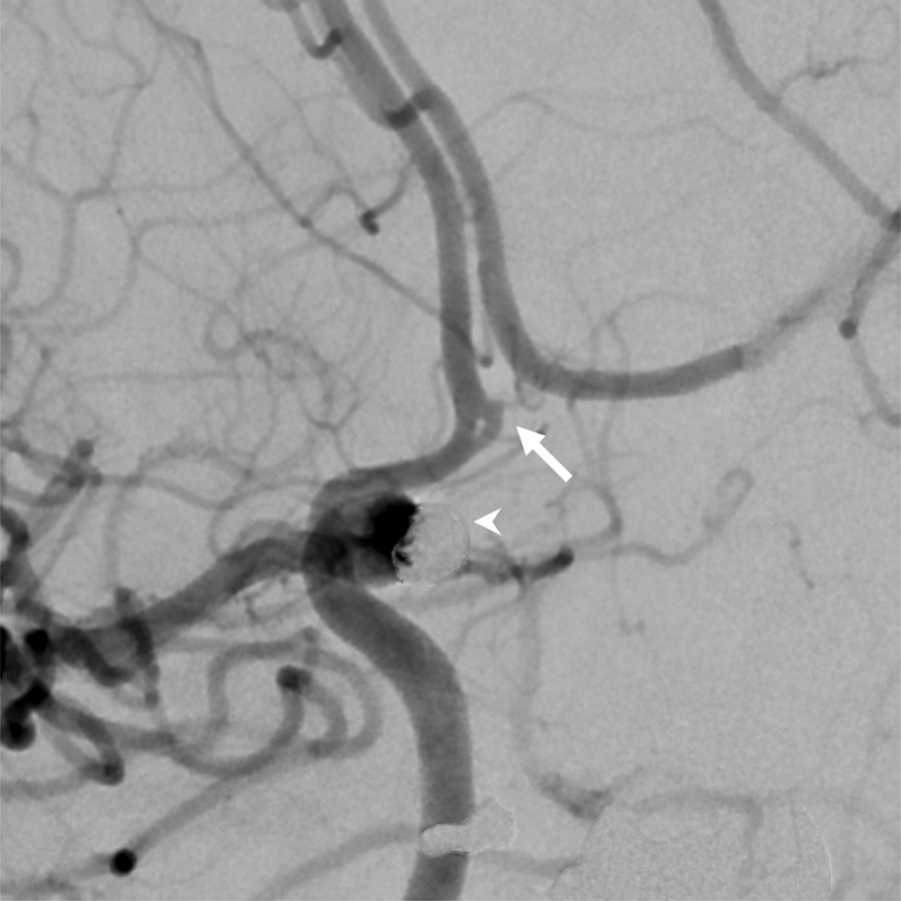
Right internal carotid angiography under left carotid artery compression showing partial duplication of the anterior communicating artery (arrow). The arrowhead indicates the paraclinoid aneurysm treated by coil embolization.

A 43-year-old woman underwent coil embolization of an unruptured right internal carotid artery aneurysm 1 year prior. She was admitted to our hospital for postoperative examinations. MRA (3-Tesla Magnetom Vida; Siemens, Erlangen, Germany) showed no recurrence of the treated aneurysm, and an arterial ring in the right part of the ACoA complex (Fig. 1). There were 2 separate ACoAs on the right side and one on the left side. Therefore, we considered this anatomical variant to be a partially duplicated ACoA. These findings were also confirmed by DSA including rotational 3-dimensional images (Fig. 2). A right internal carotid angiography under left carotid artery compression revealed no cerebral aneurysm in the ACoA complex (Fig. 3).

## Discussion

Herein, we report a rare case of a partially duplicated ACoA associated with an intracranial aneurysm. Anatomical variations in the ACA and/or ACoA complex are common and often encountered [[1], [2], [3]]. There anatomical variations include fenestration, duplication, partial duplication (such as in the present case), and double partial duplication, which show an arterial ring formation at the ACA and/or ACoA complex [2,4]. Among these variants, true fenestration of the ACoA is extremely rare, although may often be misidentified [4]. In fenestration, a single vessel divides into 2 channels with endothelial layers and muscular layers, and they join to form a single lumen on the distal side [1,2,4,6,7]. By contrast, duplication involves 2 separate vessels, as in the right part of the ACoA in our case. In a previous study of anatomical variations of the ACoA identified using computed tomography angiography, ACoA duplication was found in only 2 of 411 (0.49%) adult patients [8]. In the present case, we diagnosed a ‘partial’ duplication of the ACoA because there were 2 separate ACoAs only on the right side (Fig. 1, Fig. 2, Fig. 3). This variation is also termed an ACA A1–A2 fenestration [4]. However, this variation should not be misidentified as ACoA fenestration.

To the best of our knowledge, there are only a few reports of a partially duplicated ACoA [2,4,5]. Uchino et al. presented an example of a partially duplicated ACoA diagnosed by MRA [4]. Nedelcu et al. reported a case of a partially duplicated ACoA associated with supernumerary fronto-orbital arteries arising from the contralateral ACA, which was identified during anatomical dissection [5]. Finally, Endo et al. reported a case of a partially duplicated ACoA associated with a persistent primitive olfactory artery and an accessory middle cerebral artery, which was diagnosed by 1.5-Tesla MRA [2]. The present study provides new evidence of a partially duplicated ACoA associated with an intracranial aneurysm, which was diagnosed by 3-Tesla MRA and DSA. Partially duplicated ACoA may have other anatomical variations and/or associated aneurysms. Our case also had an intracranial aneurysm other than the ACoA complex. However, no aneurysms associated with the partially duplicated ACoA were identified, even with DSA under contralateral carotid artery compression (Fig. 3). Therefore, the clinical importance of these findings may be limited. Careful imaging assessment is important, as there are reports of mimicked aneurysms in both duplication and fenestration [1,9].

## Conclusion

We describe an extremely rare case of partial duplication of the ACoA associated with an intracranial aneurysm diagnosed by 3-Tesla MRA and DSA. This report provides additional evidence of partially duplicated ACoAs. Careful imaging assessment is important to identify this rare anatomical variant, as well as other concomitant variations and associated aneurysms.

## Ethical statement

All procedures performed in studies involving human participants were in accordance with the ethical standards of the institution and/or national research committee and with the 1964 Helsinki declaration and its later amendments or comparable ethical standards. The study was approved by the Ethics Committee of Nakamura Memorial Hospital (No. 2025013101).

## CRediT authorship contribution statement

**Hideki Endo:** Conceptualization, Methodology, Validation, Formal analysis, Investigation, Resources, Data curation, Writing - original draft, Writing - review & editing, Visualization, Project administration. **Hidetoshi Ono:** Validation, Formal analysis, Investigation, Resources, Data curation, Writing - review & editing. **Toshiichi Watanabe:** Validation, Formal analysis, Resources, Writing - review & editing. **Hirohiko Nakamura:** Supervision.

## Patient consent

Written informed consent was obtained from the patient.
